# Oligodendrogenesis after cerebral ischemia

**DOI:** 10.3389/fncel.2013.00201

**Published:** 2013-10-29

**Authors:** Ruilan Zhang, Michael Chopp, Zheng Gang Zhang

**Affiliations:** ^1^Department of Neurology, Henry Ford HospitalDetroit, MI, USA; ^2^Department of Physics, Oakland UniversityRochester, MI, USA

**Keywords:** cerebral ischemia, oligodendrocytes, oligodendrocyte progenitor cells, neural stem cells, microRNAs

## Abstract

Neural stem cells in the subventricular zone (SVZ) of the lateral ventricle of adult rodent brain generate oligodendrocyte progenitor cells (OPCs) that disperse throughout the corpus callosum and striatum where some of OPCs differentiate into mature oligodendrocytes. Studies in animal models of stroke demonstrate that cerebral ischemia induces oligodendrogenesis during brain repair processes. This article will review evidence of stroke-induced proliferation and differentiation of OPCs that are either resident in white matter or are derived from SVZ neural progenitor cells and of therapies that amplify endogenous oligodendrogenesis in ischemic brain.

## INTRODUCTION

More than 80% of stroke is ischemic stroke triggered by blockage of blood flow within major cerebral arteries by clots, which leads to infarction in white and gray matter (Dewar et al., 2003; Cui et al., 2009; Karki et al., 2009). Studies from experimental stroke and patients with stroke show that adult brain has the capability to self-repair in response to stroke. However, the spontaneous brain repair process is constrained with limited improvement of neurological outcome (Benowitz and Carmichael, 2010). Thus, stroke remains the leading cause of adult disability around the world (Demaerschalk et al., 2010).

Neurogenesis, oligodendrogenesis, angiogenesis, and astrogliosis are major brain repair processes during stroke recovery (Zhang and Chopp, 2009). Cerebral ischemia induces neurogenesis and angiogenesis in the adult human and rodent brains, which have been studied in depth (Jin et al., 2001, 2006; Zhang et al., 2001; Arvidsson et al., 2002; Parent et al., 2002; Macas et al., 2006; Minger et al., 2007; Curtis et al., 2011). In contrast, stroke-induced oligodendrogenesis in ischemic brain has not been broadly studied (Dewar et al., 2003; Pham et al., 2012). Oligodendrocytes, myelin forming cells in the central nervous system (CNS), are vulnerable to cerebral ischemia (Pantoni et al., 1996; Dewar et al., 2003). Loss of oligodendrocytes and their myelin impairs axonal function (Franklin, 2002). New oligodendrocytes are required to form myelin sheaths for sprouting axons during brain repair processes after stroke because mature oligodendrocytes do not proliferate in the adult brain and injured oligodendrocytes no longer form new myelin sheets (Gensert and Goldman, 1997; Gregersen et al., 2001; Franklin, 2002; Menn et al., 2006; Franklin and Ffrench-Constant, 2008; McTigue and Tripathi, 2008). New oligodendrocytes are derived from non-myelinating oligodendrocyte progenitor cells (OPCs; Gensert and Goldman, 1997; Franklin, 2002). Using an inducible cre-lox fate mapping strategy in adult neural progenitor cells of transgenic mice, recent studies demonstrate that OPCs originating from neural progenitor cells in the subventricular zone (SVZ) of the lateral ventricle differentiate into myelin forming oligodendrocytes under physiological and ischemic conditions (Li et al., 2010; Zawadzka et al., 2010; Zhang et al., 2011, 2012; Rafalski et al., 2013). Preclinical studies show that enhancement of endogenous oligodendrogenesis in ischemic brain by cell-based and pharmacological therapies facilitates brain repair processes and reduces neurological deficits (Li et al., 2005; Zhang and Chopp, 2009; Morris et al., 2010; Zhang et al., 2010b, 2012). These findings have led to a hope for a neurorestorative treatment of stroke which aims to manipulate endogenous neurogenesis, angiogenesis and oligodendrogenesis and thereby to enhance brain repair. In this article, we will review proliferation and differentiation of OPCs in adult rodent brain after focal cerebral ischemia and therapies that amplify endogenous oligodendrogenesis in ischemic brain. Molecular mechanisms that mediate oligodendrogenesis after stroke will also be reviewed.

## STROKE INDUCES OLIGODENDROGENESIS

Oligodendrocyte progenitor cells identified by the chondroitin sulfate proteoglycan NG2 and platelet-derived growth factor receptor α (PDGFRα) comprise 3–9% of the total cell number in the adult CNS and are the majority of proliferating cells (Dawson et al., 2003; McTigue and Tripathi, 2008). These OPCs continuously differentiate into mature myelinating oligodendrocytes throughout the gray and white matter of the adult brain (Gensert and Goldman, 1997; Franklin, 2002; McTigue and Tripathi, 2008; Fancy et al., 2011). OPCs are locally present in the corpus callosum, the striatum, and the cortex and are derived from neural progenitor cells in the SVZ (Nait-Oumesmar et al., 1999; Roy et al., 1999; Picard-Riera et al., 2002; Fancy et al., 2004; Menn et al., 2006). The SVZ contains glial fibrillary acidic protein (GFAP) expressing neural stem cells that generate intermediate progenitors (Alvarez-Buylla et al., 2008). Retroviral lineage tracking studies show that although the majority of the neural progenitor cells give rise to homogeneous neuronal progeny, some progenitor cells generate OPCs in the adult brain (Menn et al., 2006). OPCs generated from the SVZ migrate to white matter tracts of corpus callosum, fimbria fornix and striatum (Menn et al., 2006). Studies of inducible Cre recombination transgenic mice confirm Ascl1- and nestin-expressing neural stem and progenitor cells in adult brain generate OPCs (Li et al., 2010; Zhang et al., 2011, 2012). Early retroviral lineage tracking studies show that the production of OPCs by adult SVZ neural stem cells is limited (Menn et al., 2006). However, using nestin CreER; mT/mG mice in which Cre recombination leads to deletion of a membrane-targeted tomato fluorescent protein and expression of a membrane-targeted green fluorescent protein in adult neural stem and progenitor cells, Rafaski et al. (2013) recently demonstrated that a substantial fraction of OPCs are generated from adult SVZ nestin-expressing neural stem and progenitor cells under physiological condition. *In vitro*, tracking the lineage progression of primary adult SVZ neural stem cells, Ortega et al. (2013) recently demonstrated that the adult neural stem cells generated oligodendrogliogenic clones which gave raise to NG2 positive OPCs by symmetric cell division or NG2 positive OPC and GFAP positive astroglial progeny by asymmetric cells division. However, oligodendrogliogenic clones do not generate OPC and neuronal progeny. These data suggest that adult neural progenitor cells contain oligodendrogliogenic and neuronal lineages and they are distinct and not shared (Ortega et al., 2013).

Stroke acutely induces mature oligodendrocyte damage, leading to loss of myelin, which is associated with loss of axons (Pantoni et al., 1996; Dewar et al., 2003). However, during stroke recovery there is a significant increase in generation of OPCs and some of them become mature myelinating oligodendrocytes in peri-infarct gray and white matter where sprouting axons are present (Gregersen et al., 2001; Zhang et al., 2011, 2012; Ueno et al., 2012). An increase in mature myelinating oligodendrocytes observed after stroke in the adult brain likely result from new oligodendrocytes differentiated from OPCs. Studies in adult transgenic mice that express a tamoxifen-inducible form of Cre recombinase under control of an Ascl1 or nestin promoter have shown that OPCs located in the gray and white matter and OPCs from the SVZ are involved in generation of new oligodendrocytes (Zhang et al., 2011, 2012). During the first 2 weeks after stroke, a robust increase in Ascl1-expressing OPCs was observed in ischemic boundary regions of the gray and white matter. However, 2 months after stroke, the Ascl1 or nestin lineage cells in per-infarct white matter exhibited myelin sheet morphology and expressed protein components of myelin, cyclic nucleotide 3’-phosphodiesterase (CNPase) and myelin basic protein (MBP; Zhang et al., 2011, 2012). In addition to resident OPCs in white matter, stroke recruits SVZ neural progenitor cell-generated OPCs by attracting them from the SVZ to the ischemic striatum and corpus callosum (Zhang et al., 2010b, 2011, 2012). It is uncertain whether SVZ neural progenitor cells share neuronal and oligodendrocyte lineages after experimental induction of demyelination, although under physiological conditions they do not (Ortega et al., 2013). Demyelination in the corpus callosum produced by lysolecithin induces SVZ generated doublecortin (DCX) lineage neuroblasts to differentiate into OPCs (Jablonska et al., 2010). However, DCX lineage OPCs were not detected in adult ischemic brain, although stroke greatly increases DCX lineage neuroblasts (Liu et al., 2009; Zhang et al., 2009). These data indicate that stroke induces oligodendrogenesis by recruiting resident OPCs in white and gray matter and OPCs generated by SVZ neural progenitor cells and that new oligodendrocytes generated after stroke become mature myelinating oligodendrocytes. SVZ generated OPCs are present in humans after demyelination (Nait-Oumesmar et al., 2007). Stromal-derived factor 1α (SDF-1α) and vascular endothelial growth factor (VEGF) secreted by activated cerebral endothelial cells in the ischemic boundary region are likely involved in OPC migration to peri-infarct gray and white matters (Zhang et al., 2000; Imitola et al., 2004; Ohab et al., 2006; Robin et al., 2006; Hayakawa et al., 2012; Bain et al., 2013; de Castro et al., 2013). Glutamatergic inputs from damaged axons in the corpus callosum may also trigger migration of OPCs from the SVZ to peri-infarct areas (Etxeberria et al., 2010).

In addition to serving as a source to generate myelination oligodendrocytes, OPCs act as a surveillance network to detect brain injury (Hughes et al., 2013). Molecules produced by reactive astrocytes and microglia after brain injury trigger OPC proliferation and regulate OPC differentiation (Moore et al., 2011; Miron et al., 2013). For example, insulin-like growth factor-1 (IGF-1) and bone morphogenic proteins (BMPs) secreted by astrocytes promotes and inhibit, respectively, the generation of myelinating oligodendrocytes (Moore et al., 2011). Transformation of pro-inflammatory (M1) microglia and macrophages to anti-inflammatory (M2) drives OPCs to differentiate into mature oligodendrocytes in a model of focal demyelination (Miron et al., 2013). However, the role of such cross-talk in mediating stroke-induced oligodendrogenesis has not been investigated.

## THERAPIES ENHANCE ENDOGENOUS OLIGODENDROGENESIS IN THE ISCHEMIC BRAIN

Stroke increases OPC generation in the SVZ, yet endogenous oligodendrogenesis from SVZ neural stem and progenitor cells in response to stroke is limited. Emerging preclinical studies show that cell and pharmacological based therapies initiated at days after stroke enhance endogenous oligodendrogenesis and axonal outgrowth (Li et al., 2005; Zhang and Chopp, 2009; Morris et al., 2010; Zhang et al., 2010b, 2012). Erythropoietin (EPO) regulates neural stem and progenitor cell function through interaction with its receptor EPOR in the adult SVZ (Shingo et al., 2001; Tsai et al., 2006; Chen et al., 2007; Wang et al., 2007). Administration of recombinant human EPO (rhEPO) 24 h after stroke induced sustained OPC proliferation in the peri-infarct white matter and the SVZ (Zhang et al., 2010b). Moreover, rhEPO treatment substantially amplified myelinating oligodendrocytes and increased myelinated axons in peri-infarct white matter (Jiang et al., 2006; Zhang et al., 2010b). Thymosin β4 (Tβ4) is a G-actin binding protein (Goldstein et al., 2005). Administration of Tβ4 24 h after stroke robustly increased NG2 positive OPCs in the SVZ and mature myelinating oligodendrocytes and OPCs in the peri-infarct striatum and corpus callosum 2 months after stroke (Morris et al., 2010). Treatment of stroke with cerebrolysin, a mixture of neurotrophic peptides, amplified generation of OPCs in the SVZ and mature oligodendrocytes in white matter of the peri-infarct region (Zhang et al., 2010a, 2013). Furthermore, administration of mesenchymal stromal cells (MSCs) even at 7 days after stroke substantially increased NG2 positive OPCs in the SVZ and MBP positive oligodendrocytes in the peri-infarct striatum and corpus callosum 4 months after stroke (Li et al., 2005). The effect of MSCs on enhancement of oligodendrogenesis appears specific because the treatment with MSCs significantly reduced GFAP positive astrocytes in peri-infarct regions (Li et al., 2005). These data indicate that cell and pharmacological based therapies amplify stroke-induced oligodendrogenesis. Aging reduces oligodendrocytes in rodent and human brains (Sim et al., 2002; Pelvig et al., 2008; Shen et al., 2008a,b). By tracking progeny of SVZ nestin lineage neural progenitor cells in ischemic brain of transgenic mice at age of 12 months, a study shows that stroke increased nestin lineage OPCs and oligodendrocytes and that administration of sildenafil, a potent phosphodiesterase type 5 (PDE5) inhibitor, further augmented nestin lineage OPCs and oligodendrocytes in peri-infarct corpus callosum and striatum (Zhang et al., 2012). These data suggest that even in aged animals, oligodendrogenic potential is present in SVZ neural progenitor cells in response to stroke and the treatment. More importantly, increases in new oligodendrocytes are closely associated with augmentation of sprouting axons in peri-infarct areas, which may contribute to functional recovery after stroke, although further studies are warranted to demonstrate newly generated oligodendrocytes myelinate axons in peri-infarct regions.

## SIGNALING PATHWAYS MEDIATE OLIGODENDROGENESIS IN THE ISCHEMIC BRAIN

Sonic hedgehog (Shh) is a member of the family of the hedgehog proteins and binds to the transmembrane receptor protein, patched (ptc), which, in the absence of Shh, exerts an inhibitory effect on the seven transmembrane receptor smoothened (Smo; Ingham and McMahon, 2001; Gutierrez-Frias et al., 2004). In the canonical way, binding of Shh to ptc blocks the inhibitory effect of ptc on Smo. Once activated, Smo induces complex series of intracellular reactions that targets the Gli family of transcription factors (Ruiz i Altaba et al., 2002). In addition to neurogenesis, the Shh signaling pathway regulates oligodendrogenesis by inducing a basic helix-loop-helix transcription factor Olig2 that is required for specification of NG2 positive OPCs and mediates OPC differentiation (Arnett et al., 2004; Ligon et al., 2004, 2006; de Castro et al., 2013; Ferent et al., 2013). In the adult brain, Olig2-expressing neural progenitor cells in the SVZ give rise to OPCs that migrate to the white matter (Arnett et al., 2004; Ligon et al., 2006; Menn et al., 2006). Stroke upregulated Shh signal in SVZ neural progenitor cells and blockage of the Shh pathway with cyclopamine, a specific inhibitor of Smo, suppressed stroke-induced neural progenitor cell proliferation and attenuated EPO-increased neural progenitor cell proliferation (Wang et al., 2007; Liu et al., 2013a). Furthermore, administration of cyclopamine to ischemic animals abolished cerebrolysin-enhanced oligodendrogenesis (Zhang et al., 2013). Consistent with stroke, in a model of focal demyelination induced by lysolecithin in the corpus callosum of adult mice, the blocking of Shh signaling with its physiological antagonist, hedgehog interacting protein, led to a decrease of OPC proliferation and differentiation (Ferent et al., 2013). Together, these data suggest that the Shh pathway in SVZ neural progenitor cells plays an important role in mediating oligodendrogenesis in the ischemic brain.

Wnt signaling in adult neural progenitor cells regulates oligodendrogenesis (Rafalski et al., 2013). Overexpression of Wnt3 in SVZ neural progenitor cells results in a substantial and selective increase of PDGFRα positive OPCs in adult mouse brain (Rafalski et al., 2013). However, the canonic Wnt signaling pathway negatively regulates OPC differentiation (Fancy et al., 2011). The relevance of Wnt signaling to ischemia-induced oligodendrogenesis remains to be established.

Phosphatidylinositol 3-kinase (PI3K) and its downstream target, Akt, affect multiple cellular functions such as cell survival,proliferation, and differentiation (Vojtek et al., 2003). Mitogen-activated protein kinases (MAPKs) belong to families of Ser/Thr-specific kinases activated by extracellular stimuli through protein phosphorylation (Rubinfeld and Seger, 2005). Extracellular signal-regulated kinases (ERKs), ERK1 and ERK2, are MAPKs (Rubinfeld and Seger, 2005). The PI3K/Akt, p38MARK and ERK1/2 signals are involved in OPC differentiation (Chew et al., 2010; Fyffe-Maricich et al., 2011; Santra et al., 2012; Rafalski et al., 2013). Inhibition of either p38 MAPK signaling with the inhibitor SB202190 or PI3K with the inhibitor LY294002 significantly reduces adult neural progenitor cells generated OPCs (Santra et al., 2012; Rafalski et al., 2013). Inactivation of p38MAPK with the inhibitor SB202190 in adult neural progenitor cells activated ERK1/2 and abolished Tβ4-increased OPCs (Santra et al., 2012). Therefore, activation of PI3K/Akt and p38MAPK signals and cross-talk between p38MAPK and ERK signals in neural progenitor cells appear to be important in regulating generation of OPCs.

## MICRORNAs MEDIATE PROCESSES OF OLIGODENDROGENESIS AFTER STROKE

MicroRNAs (miRNAs), small non-coding RNAs, regulate neural stem cell function and play a pivotal role in controlling processes of OPC generation and differentiation (He et al., 2012). Dicer is an endoribonuclease that cleaves double-stranded RNA and pre-miRNA into short double-stranded RNA (Krol et al., 2010). During development, disruption of miRNA biogenesis by conditional ablation of Dicer in nestin lineage neural progenitor cells results in neural progenitor cell death and abnormal neuronal OPC differentiation (Kawase-Koga et al., 2009). Conditional deletion of Dicer in Olig2 lineage cells led to impairment of OPC differentiation (Dugas et al., 2010; Nave, 2010; Zhao et al., 2010), whereas ablation of Dicer in proteolipid protein (PLP) lineage oligodendrocytes resulted in dysmyelination (Shin et al., 2009). These data suggest that in addition to neural progenitor cells, miRNAs are required for maintaining OPCs in the undifferentiated state and for preserving myelin in mature oligodendrocytes. Indeed, studies on miRNA profiles and functions show that OPCs and oligodendrocytes express distinct sets of miRNAs (Dugas et al., 2010; Nave, 2010; Zhao et al., 2010). For example, over expression of miR-219 and miR-338 in OPCs promoted oligodendrocyte differentiation by repressing targeting genes including PDGFRa, Sox6, Zfp238, FoxJ3, and Hes5 (Dugas et al., 2010; Zhao et al., 2010).

During development, the miR17-92 cluster, a cluster of seven miRNAs, regulates processes of proliferation and survival of CNPase-expressing oligodendrocytes via upregulation of PTEN and thus inactivation of Akt (Budde et al., 2010). The adult SVZ neural progenitor cells expressed the miR17-92 cluster and stroke substantially upregulated this cluster expression (Liu et al., 2011, 2013a,b). Attenuation of members of the miR17-92 cluster, miR18a and miR19a, or elevation of this cluster in adult SVZ neural progenitor cells suppressed or enhanced neural progenitor cell proliferation, respectively, via alteration of PTEN protein levels (Liu et al., 2013a). Thus, the miR17-92 cluster may regulate processes of oligodendrogenesis in adult brain. The Shh signaling pathway functionally interacts with the miR-17-92 cluster in neural progenitor cells in mediating cell proliferation (Northcott et al., 2009; Uziel et al., 2009). *In vitro* and *in vivo* studies show that activation and suppression of the Shh signaling pathway down- and up- regulated, respectively, expression of the miR17-92 cluster in SVZ neural progenitor cells under non-ischemic and ischemic conditions (Liu et al., 2013a,b).

In addition, miR-9 and miR-200b are likely involved in stroke-induced oligodendrogenesis by targeting serum response factor (SRF; Buller et al., 2012). Stroke considerably downregulated miR-9 and miR-200b in white matter. Overexpression of miR-9 and miR-200 in OPCs suppressed SRF expression and inhibited OPC differentiation (Buller et al., 2012). Collectively, these findings demonstrate that miRNAs are involved in processing stroke-induced oligodendrogenesis.

## HISTONE DEACETYLASES AND STROKE-INDUCED OLIGODENDROGENESIS

Classes I and II histone deacetylase (HDAC) activity is required for oligodendrocyte differentiation during brain development (Shen and Casaccia-Bonnefil, 2008; Shen et al., 2008a,b). Pharmacological inhibition of HDAC activity and conditional ablation of HDAC1 and HDAC2 in the oligodendrocyte lineage cells lead to reduction of OPCs and mature oligodendrocytes (Shen and Casaccia-Bonnefil, 2008; Shen et al., 2008a,b; Ye et al., 2009). There are few studies that have examined the role of classes I and II HDACs in mediating processes of oligodendrogenesis in ischemic brain. Stroke increased HDAC 1 and HDAC2 proteins in OPC nuclei and cytoplasmic HDAC4 proteins in OPCs, which was accompanied by reduction of the acetylation levels of histones H3 and H4 (Kassis et al., 2013). Interestingly, treatment of stroke with valproic acid, a pan HDAC inhibitor, considerably increased OPCs and new oligodendrocytes in the adult rat (Liu et al., 2012). These data suggest that HDACs are involved in stroke-induced oligodendrogenesis. The sirtuins, a family of NAD-dependent histone deacetylases, regulate crucial metabolic pathways and are linked to lifespan (Penner et al., 2010; Yu and Auwerx, 2010). Inactivation of SIRT1 in SVZ neural progenitor cells expanded OPCs, which was mediated by activation of Akt and p38 MAPK signaling (Rafalski et al., 2013). However, additional studies are needed to investigate the specific roles of individual HDACs and SIRT1 in proliferation and differentiation of OPCs during adult brain repair.

## SUMMARY

Stroke induces oligodendrogenesis. OPCs resident in white matter and OPCs derived from neural progenitor cells contribute to generation of mature myelination oligodendrocytes that interact with axons and astrocytes during post stroke brain remodeling. Potential mechanisms underlying stroke-induced oligodendrogenesis are emerging. Recent studies show that in addition to facilitating salutatory conduction, myelination in adult brain contribute to maintaining axonal integrity, neural plasticity and circuitry function (Fields, 2008; Nave, 2010; Fancy et al., 2011; Zatorre et al., 2012; Young et al., 2013). It is essential for future studies to investigate mechanisms that temporally and spatially coordinate controlling oligodendrogenesis at multiple stages, and to study relevance of remyelination by oligodendrogenesis to neuronal circuitry, which will greatly enhance the development of new therapies for stroke and other demyelination diseases.

## Conflict of Interest Statement

The authors declare that the research was conducted in the absence of any commercial or financial relationships that could be construed as a potential conflict of interest.
